# Biodegradation, Decolorization, and Detoxification of Di-Azo Dye Direct Red 81 by Halotolerant, Alkali-Thermo-Tolerant Bacterial Mixed Cultures

**DOI:** 10.3390/microorganisms10050994

**Published:** 2022-05-09

**Authors:** Islam M. Kamal, Nourtan F. Abdeltawab, Yasser M. Ragab, Mohamed A. Farag, Mohammed A. Ramadan

**Affiliations:** 1 Department of Microbiology and Immunology, Faculty of Pharmacy, Cairo University, Cairo 11562, Egypt; yasser.ragab@pharma.cu.edu.eg (Y.M.R.); mohamed.abdelhalim@pharma.cu.edu.eg (M.A.R.); 2 Department of Pharmacognosy, Faculty of Pharmacy, Cairo University, Cairo 11562, Egypt; mohamed.farag@pharma.cu.edu.eg; 3 Department of Chemistry, School of Sciences and Engineering, American University in Cairo, New Cairo 11835, Egypt

**Keywords:** biodegradation, biodegradation genes, wastewater, biodegradative pathways, metabolite identification, azo dye, detoxification, Direct Red 81 (DR81), azoreductase, mixed bacterial cultures

## Abstract

Azo dyes impact the environment and deserve attention due to their widespread use in textile and tanning industries and challenging degradation. The high temperature, pH, and salinity used in these industries render industrial effluent decolorization and detoxification a challenging process. An enrichment technique was employed to screen for cost-effective biodegraders of Direct Red 81 (DR81) as a model for diazo dye recalcitrant to degradation. Our results showed that three mixed bacterial cultures achieved ≥80% decolorization within 8 h of 40 mg/L dye in a minimal salt medium with 0.1% yeast extract (MSM-Y) and real wastewater. Moreover, these mixed cultures showed ≥70% decolorization within 24 h when challenged with dye up to 600 mg/L in real wastewater and tolerated temperatures up to 60 °C, pH 10, and 5% salinity in MSM-Y. Azoreductase was the main contributor to DR81 decolorization based on crude oxidative and reductive enzymatic activity of cell-free supernatants and was stable at a wide range of pH and temperatures. Molecular identification of azoreductase genes suggested multiple *AzoR* genes per mixed culture with a possible novel azoreductase gene. Metabolite analysis using hyphenated techniques suggested two reductive pathways for DR81 biodegradation involving symmetric and asymmetric azo-bond cleavage. The DR81 metabolites were non-toxic to *Artemia salina* nauplii and *Lepidium sativum* seeds. This study provided evidence for DR81 degradation using robust stress-tolerant mixed cultures with potential use in azo dye wastewater treatment.

## 1. Introduction

Rapid industrialization poses a great challenge for the efficient removal of the complex and toxic industrial eco-pollutant wastes, especially dye-contaminated industrial effluents [1,2,3]. There are approximately 100,000 known dyes worldwide, with a consumption rate of 10,000 tons/year, and about 5–10% are released in industrial effluent, where azo dyes represent 60% of the used dyes [4,5,6]. Synthetic azo dyes are used in textile dyeing, paper printing, leather dyeing, color photography, and as additives in petroleum industries. Moreover, synthetic dyes are more stable than natural dyes, which is one of the reasons behind being more recalcitrant to dye degradation. [4,5,7]. Textile and tanning industrial effluents are treated to remove any traces of synthetic azo dyes that might accumulate in the environment, causing phytotoxicity as well as damage to aquatic ecosystems [8]. Further, these contaminated effluents are released under harsh conditions during industrial processing and are characterized by challenging physicochemical parameters, including high chemical oxygen demand (COD), biological oxygen demand (BOD), high temperature, salts, total suspended and dissolved solids, and alkalinity [9,10]. Moreover, synthetic azo dyes impose serious health hazards, such as carcinogenicity and mutagenicity [11]. Consequently, governments set laws and legislations to achieve safe disposal of xenobiotics in such effluents to guard against the adverse effects of industrial effluents containing azo dyes and their degradation products on aquatic and terrestrial lives to prevent pollution of natural ecosystems [12]. Therefore, to comply with governmental legislation, several approaches for the treatment and detoxification of industrial effluent wastewater have been explored using physical, chemical, and biological systems.

Biological wastewater treatment approaches have several advantages, including lower cost, being ecofriendly, and production of a relatively lower quantity of sludge than physical and chemical approaches [13,14]. The biodegradation of dyes involves the use of microbes indigenous to their contaminated habitats, such as fungi, algae, and bacteria, where bacteria have the advantage of rapid growth under different environmental conditions [9,15]. Moreover, mixed bacterial cultures are more efficient than pure cultures in decolorization with a higher rate and complete degradation with multiple enzymes that attack the dye molecule at different sites [16]. Biodegradation of wastewater occurs by dye-degrading oxidoreductive enzymes, such as tyrosinases, laccases, peroxidases, veratryl alcohol oxidase, and azoreductases [17,18]. Azoreductases are one of the most common enzymes found in many bacteria, such as *Pseudomonas* sp., *Enterococcus faecalis* YZ 66, and *Bacillus* sp. [19,20,21]. Azoreductases are classified into various classes based on co-enzyme requirements, flavin-dependent and independent [16]. 

Biological degradation of colored textile and tanning effluents containing azo dyes by bacteria usually involves two steps for bond cleavage. The first step is azo bond (N=N) reduction via azoreductases resulting in toxic aromatic amine metabolites, which are further degraded in the second step leading to less toxic metabolites or complete mineralization [21,22]. Hence, to assess the effectiveness of the biodegradation system, it is important to check the toxicity of both the untreated dye and its produced metabolites after dye degradation to ensure the safety of the applied biodegradative method. Phytotoxicity on a plant model such as water cress (*Lepidium sativum*) and acute toxicity on brine shrimp nauplii (*Artemia salina*) are among the most used methods for toxicity evaluation [14,23,24]. 

Among the known toxic azo dyes is the diazo sulfonated hydrophilic compound Direct Red 81 (DR81), which consists of two azo groups (–N=N–) linked by aromatic rings [21]. Other dyes share the same nucleus as azo dye DR81 (R-N=N-R’), where one or more azo bonds (N=N) along with aryl groups (R and R’) form the main nucleus. Dyes that share the same nucleus as DR81 include reactive black 5, direct blue 1, and direct black 22 [25]. The chromophore part of the DR81 structure contains the conjugated chemical groups, such as aromatic rings, azo bonds, and carbonyl groups. In addition, there are other functional groups responsible for the auxochrome part in the molecule, such as amine, hydroxyl, sulphonic, and carboxyl groups. The auxochrome functional groups are responsible for the dye charge, solubility, and cellulose binding affinity. Direct dyes such as DR81 represent more than half of the dyes used in textile and pulp industries as they have sulphonic acid groups making them more water-soluble and easier to bind to fibers [26]. This study focused on DR81 biodegradation as it poses an environmental hazard due to its high molecular weight and being disodium salt; therefore, it is highly water-soluble, making it difficult to remove compared to mono azo dyes [16]. Therefore, this study aimed to search for robust decolorizing mixed bacterial culture for the biological treatment of the recalcitrant DR81 azo dye with emphasis on the azoreductase enzyme responsible for biodegradation in addition to prediction of the possible pathway of DR81 degradation.

## 2. Materials and Methods

### 2.1. Chemicals and Dyestuff

Direct Red 81 (DR81), also known as disodium 7-benzamido-4-hydroxy-3-[[4-[(4-sulphonatophenyl)azo]phenyl]azo]naphthalene-2-sulphonate, its empirical formula: C29H19N5Na2O8S2, *n*-propanol, veratryl alcohol, MgSO_4_·7H_2_O, CaCl_2_, FeSO_4_·7H_2_O, and flavin mononucleotide (FMN) were all purchased from Sigma Aldrich (St. Louis, MO, USA). β-Nicotinamide adenine dinucleotide reduced form (NADH), and methanol high-performance liquid chromatography (HPLC) grade were purchased from Fisher Chemical (Fisher Scientific, Hampton, NH, USA). All other chemicals were of high purity analytical grade purchased from El Gomhoreya Co. (Cairo, Egypt).

### 2.2. Microorganisms, Culture Conditions and Acclimatization

#### 2.2.1. Sample Collection

Sixty-seven samples from textile and tanning wastewater (35), soil from dye-contaminated sites (7), and swabs from waste on dyeing machines (24) were collected from the tanning industrial zone at Ain el Sira and textile factories in October City industrial zone, Cairo, Egypt. Moreover, one brewer yeast sample was obtained from a local bakery in Ain el Sira, Cairo, Egypt.

#### 2.2.2. Development and Enrichment of Culturable Mixed Microbial Cultures

**Figure 1:**
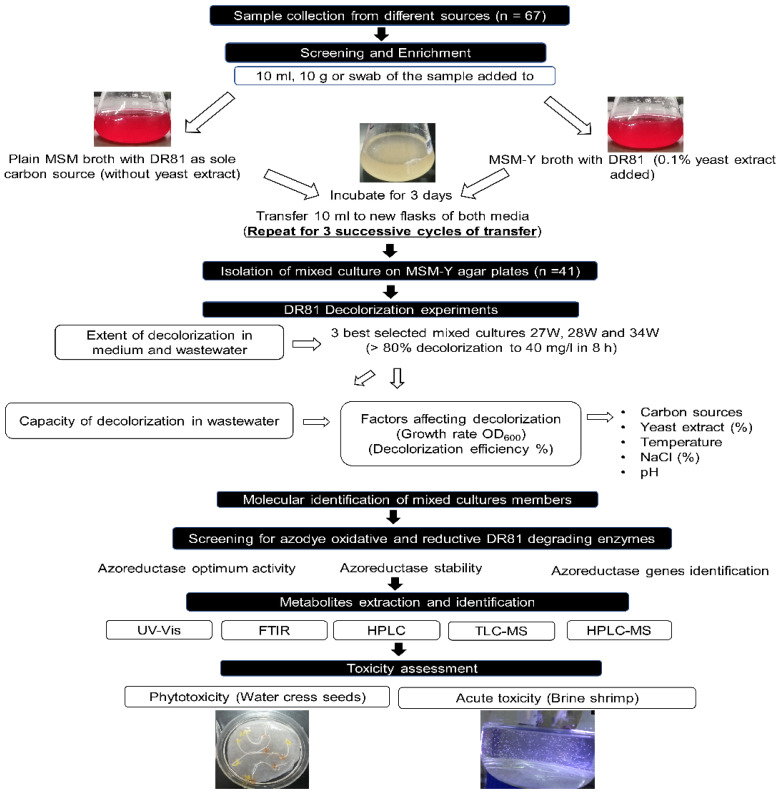
**Experimental design and workflow for screening, enrichment, and isolation of decolorizing mixed cultures capable of biodegrading and detoxifying DR81 azo dye**. Isolation of DR81 decolorizing mixed cultures via the enrichment technique, followed by evaluation of the extent and capacity of decolorization. Factors affecting decolorization were then determined, and members of potential mixed cultures were molecularly identified using *16s rRNA* gene sequencing. Degrading enzymes were then determined, and potential metabolites were identified. Finally, phytotoxicity and acute toxicity of decolorized supernatants were evaluated. Abbreviations: mineral salt medium (MSM), Direct Red 81 (DR81), MSM with 0.1% yeast extract (MSM-Y), ultraviolet-visible spectral scan analysis (UV-Vis), Fourier transform infrared spectroscopy (FTIR), high-performance liquid chromatography/UV detector (HPLC), thin-layer liquid chromatography/mass spectroscopy (TLC-MS), high-performance liquid chromatography/mass spectroscopy (HPLC-MS).

Microbial cultures capable of degrading azo dyes were screened and selected using enrichment methods modified from Khalid et al. [27], as outlined in Figure 1. Briefly, 10 mL (or 10 g) of collected samples were added to 40 mL of plain mineral salt medium (MSM) prepared according to Hashem et al. [28] supplemented with 40 mg/L DR81 dye acting the as sole carbon source. The plain MSM was composed of 4 g K_2_HPO_4_, 4 g KH_2_PO_4_, 2 g (NH_4_)_2_SO_4_, 0.5 g MgSO_4_·7H_2_O, 0.01 g CaCl_2_, 0.01 g FeSO_4_·7H_2_O; all components were calculated per liter of distilled water and adjusted to pH 7. The second medium used was MSM supplemented with 0.1% yeast extract (MSM-Y) with 40 mg/l DR81 dye, where the yeast extract (1 g) per liter was added to obtain MSM-Y. For both media, the cultures were incubated at 30 °C statically for three days. After 3 days of incubation, 10 mL aliquot was withdrawn and added to fresh medium of MSM or MSM-Y containing 40 mg/L DR81 dye and incubated for another 3 days. For acclimatization, this aliquoting cycle was repeated three successive times for a total of nine days. After the 3rd cycle, an aliquot of 0.5 mL was withdrawn from samples showing complete decolorization and spread onto MSM-Y-DR81 agar plate and incubated at 30 °C till visible colonies appeared. The MSM-Y-DR81 agar was obtained by adding 2% agar to MSM-Y broth containing 40 mg/L DR81. The obtained colonies of mixed bacterial cultures from MSM-Y-DR81 agar plates were sub-cultured in MSM-Y broth with 40 mg/L DR81; broth of mixed cultures was added to glycerol (50%) and stored at −80 °C freezer and used for all subsequent studies.

### 2.3. Decolorization Studies

#### 2.3.1. Extent of Decolorization in MSM-Y and Real Wastewater

DR81 with a concentration of 40 mg/L was supplemented with 100 mL of MSM-Y, sterile wastewater (SWW), and non-sterile wastewater (NSSW). Real wastewater was obtained from the tanning industrial zone at Ain el Sira, Cairo, Egypt. All flasks were inoculated with 6% overnight cultures (OD_600_ 1.7 ± 0.2) of decolorizing mixed cultures. Incubation was performed statically at 30 °C. A total of 3 mL were withdrawn at 2, 4, 6, 8, and 24 h intervals, centrifuged at 4800× *g*, and percentage decolorization was calculated at ʎ_max_ 511 for DR81 according to Equation (1) based on Sahasrabudhe, Saratale, Saratale, and Pathade [19]:

(1) $$\%\;\mathrm{Decolorization}=\left[\frac{\;\mathrm{Initial}\;\mathrm{absorbance}\;-\;\mathrm{Observed}\;\mathrm{absorbance}\;}{\mathrm{Initial}\;\mathrm{absorbance}}\right]\times 100$$

Changes in pH were also recorded. The best 3 mixed cultures 27W, 28W, and 34W showing ≥80% decolorization at 8 h were chosen for further testing. The three selected mixed cultures were also tested for possible dye adsorption by checking the color of the pellets after centrifugation of the decolorized suspensions. Moreover, single colonies from the selected mixed cultures were picked, streaked onto MSM-Y-DR81 agar plates containing 40 mg/L DR81 until pure isolates were obtained, and single isolated to check their decolorization ability. Abiotic controls were performed on MSM-Y, SWW, and NSWW containing 40 mg/L DR81 dye. The physicochemical parameters of NSWW before and after decolorization by the selected mixed cultures were tested at the MicroAnalytical Center, at the faculty of science, Cairo University, using standard technical methodologies, and the results are demonstrated in Table S1. The basic characteristics of wastewater, including physical characteristics of pH, total dissolved solids (TDS), and total suspended solids (TSS), were determined using appropriate metered electrodes. Chemical and biological characteristics of wastewater were also analyzed, including levels of phenols, ammonia, nitrate, total nitrogen, carbonate, bicarbonate, phosphate, chemical oxygen demand (COD), and biological oxygen demand (BOD) using NANOCOLOR 500D photometer system for the analysis of wastewater (Macherey-Nagel, Düren, Germany) according to Eraqi et al. [29].

#### 2.3.2. Effect of Initial Dye Concentration on Decolorization in Real Wastewater

Increasing concentrations of DR81 azo dye (10, 40, 80, 200, 400, and 600 mg/L) were used to access the capacity of the selected mixed cultures on decolorization in NSWW. All flasks containing NSWW were inoculated with 6% overnight cultures (OD_600_ 1.7 ± 0.2) of decolorizing mixed cultures, incubated statically at 30 °C. Three milliliters were withdrawn at 2, 4, 6, 8, and 24 h intervals, centrifuged at 4800× *g*, and percentage decolorization was calculated at ʎ_max_ 511 for DR81 as described above in Section 2.3.1 [19]. Abiotic controls were performed using NSWW containing DR81 dye.

### 2.4. Factors Affecting DR81 Decolorization by the Selected Mixed Bacterial Cultures

The effect of different physico-chemical parameters on DR81 decolorization by the selected mixed cultures was tested in MSM-Y broth. All flasks were inoculated with 6% overnight cultures (OD_600_ 1.7 ± 0.2) of decolorizing mixed cultures and incubated statically at 30 °C. A total of 3 mL was withdrawn at different time intervals, centrifuged at 4800× *g,* and percentage decolorization was calculated at ʎ_max_ 511 for DR81 as described above in Section 2.3.1. Abiotic control was performed in all conditions. Different organic and inorganic carbon sources were 5 mM of glucose, sucrose, lactose, glycerol, sodium citrate, and sodium acetate. The yeast extract (YE) concentration was tested (0.1%, 0.3%, 0.5%, 0.7%, and 1%). The challenging factors to test the efficiency of decolorization were temperature (16, 25, 30, 40, 50, and 60 °C), pH (5, 6, 7, 8, 9, and 10), and salinity (1%, 3%, and 5% NaCl). Inoculum sizes tested were 6%, 10%, and 20%. The effect of agitation and static conditions were also tested. Changes in decolorization after 2 and 4 h were plotted.

### 2.5. Molecular Identification of Pure Isolated Members of the Selected Mixed Bacterial Cultures Using Sanger Sequencing of the 16S rRNA Gene Full Length

Single colonies from each mixed culture were picked and streaked on MSM-Y-DR81 agar plates with multiple subcultures until pure colonies appeared. Pure colonies obtained from selected mixed bacterial cultures were subjected to molecular identification through sequencing of their 16S rRNA gene. The universal primers used were 8F 5′AGAGTTTGATCCTGGCTCAG3′ and 1492 R 5′CGGTTACCTTGTTACGACTT’3 at 57–59 °C with a product size 1500 bp [21]. PCR amplification was performed using GoTaq^®^ G2 Flexi DNA polymerase (Promega, Madison, WI, USA) according to the following steps: Initial denaturation of 94 °C for 2 min, followed by 30 cycles of denaturation at 94 °C for 1 min, annealing at 57–59 °C for 1 min, extension at 72 °C for 1 min/1 kb, and final extension of 72 °C for 5 min. Amplification products were run using gel electrophoresis and then purified by QIAquick^®^ PCR Purification kit or QIAquick gel extraction kit (Qiagen, Germany). Next, DNA sequencing was performed with an automated DNA sequencer (ABI 3130XL; Applied Biosystems Instrument, Carlsbad, CA, USA) using the BigDye Terminator v3.1 cycle sequencing kit performed at the Macrogen sequencing facility (Macrogen, Korea). The sequencing data were analyzed using SeqMan Pro v10.0.1 (DNASTAR, Madison, WI, USA) by assembling the forward and reverse reads into a consensus sequence. The sequences were trimmed to remove the noise, then aligned using the BLAST server on the NCBI database to check for the best matching sequence using the (nr/nt) database.

### 2.6. Screening for Azo Dye Degrading Enzymes in the Selected Mixed Bacterial Cultures

#### 2.6.1. Preparation of the Cell-Free Supernatant

The tested mixed cultures were grown in 100 mL MSM-Y containing 40 mg/L DR81. After 2 h, the cultures were centrifuged at 4800× *g* for 15 min at 4 °C, the pellets were washed 3 times, and resuspended in 50 mM potassium phosphate buffer pH 7.4. The cells were disrupted using a probe sonicator followed by centrifugation at 4800× *g* for 15 min at 4 °C, and the cell-free supernatants (CFS) were used for screening of enzymatic activities.

#### 2.6.2. Screening for Reductive and Oxidative Degrading Enzymes

The azoreductase assay was performed according to Kalyani et al. [30] with some modifications. The 1 mL reaction mixture contained 20 µM DR81 (5 µL of 2 mM stock), 100 µM FMN (10 µL of 10 mM stock), 335 µL 50 mM potassium phosphate buffer pH 7.4, and 600 µL CFS; the reaction was initiated by the addition of 5 mM NADH. The decrease in absorbance of DR81 at OD_511_ was observed. One unit of azoreductase activity was defined as 1 µM of DR81 reduced per minute per milligram total protein (ε = 0.02064 µM^−1^ cm^−1^), as calculated from the calibration curve (Figure S1a).

Laccase activity was tested through the oxidation of syringaldazine (ε = 0.065 µM^−1^ cm^−1^) at OD_525_ [31]. Lignin peroxidase assay was performed by monitoring the propanaldehyde formation (ε = 0.00002 µM^−1^ cm^−1^) at OD_300_. Veratryl alcohol oxidase activity was determined by veratraldehyde formation (ε = 0.0093 µM^−1^ cm^−1^) at room temperature measured at OD_310_ [32]. Total protein concentration for the CFS was determined by the Bradford method using bovine serum albumin as a standard [33].

#### 2.6.3. Azoreductase Optimum Activity and Stability Testing

Different factors were tested to obtain the optimum conditions for enzymatic activity in the CFS of the selected mixed cultures. The parameters included temperature (25–100 °C), pH (4–11), NADH concentration (1, 3, 5, and 10 mM), total protein concentration of the crude enzyme (0.3, 0.6, and 0.9 mg/mL), and FMN dependence. Optimum azoreductase activity was tested by changing one parameter in each experiment and performing the enzymatic activity as described in Section 2.6.2. To test the thermal and pH stability, azoreductase stability was tested according to Ooi et al. [34] with slight modifications where the residual activity was measured after exposure of the crude enzyme CFS for 1 h to a certain temperature (25–70 °C) or pH (4–11). The used buffers for different pH in optimum activity and stability experiments were 0.1 M acetate buffer pH 4–5, 0.1 M phosphate buffer pH 6–7, 0.1 M Tris-HCl buffer pH 8–9, and 0.1 M NaOH/NaHCO_3_ buffer pH 10–11. The control was performed from the CFS preserved on ice with 0.1 M phosphate buffer pH 7.4 without any pH exposure.

### 2.7. Identification of Azoreductase Genes among the Selected Mixed Bacterial Cultures

The primers were designed to identify the possible azoreductase genes in the mixed bacterial culture (Table S2). The primers were either species-specific or degenerate and designed based on previously identified bacteria by 16S *rRNA* gene sequencing (Table 1). The primers were designed using the NCBI Primer-BLAST tool [35] and the IDT-DNA PrimerQuest™ Tool (Integrated DNA Technologies, Inc., Coralville, IA, USA). Briefly, azoreductase protein sequences produced by different species were searched using TBLASTN from the NCBI. These sequences were retrieved to the corresponding nucleotide sequences from the Genbank RefSeq database (https://www.ncbi.nlm.nih.gov/nucleotide/, accessed on 29 January 2020). For each bacterial species, BLASTN was used to find and align all possible homologous genes from other members of the same species. The primers were designed manually by searching the most conserved nucleotide sequences among the aligned azoreductase sequences. Afterward, the primer melting temperatures, and probability of hairpin, homo, and heterodimer formations were checked by the OligoAnalyzer tool on the IDT-DNA website (https://eu.idtdna.com/calc/analyzer, accessed on 29 January 2020). The designed primers were checked for specificity using in silico PCR [36]. PCR amplification conditions, gel electrophoresis, PCR product purification, and sequencing were performed as mentioned under Section 2.5, and annealing temperatures were according to Table S2. A phylogenetic tree was constructed using CLC main work bench 5 based on DNA sequencing.

### 2.8. Metabolites Extraction and Isolation

To predict the biodegradative pathway, metabolites from the selected mixed bacterial cultures were extracted using 100 mL culture. The aliquoted cultures were centrifuged at 4800× *g* for 15 min at 4 °C, and supernatants were chromatographed on a Diaion HP-20 resin column (2 D × 24 L cm) packed with 20 g (Supelco, Bellefonte, PA, USA) according to Elbanna et al. [37]. Elution was performed with 200 mL of 100% distilled water followed by 200 mL of 100% methanol. The water fraction was discarded, and the methanol fraction was evaporated by a rotary evaporator at 40 °C to dryness. The pellets were resolubilized in 3 mL of HPLC grade 100% methanol, and the resolubilized methanolic fraction was further used for metabolite analysis.

#### 2.8.1. Spectroscopic Determination

The degradation was assessed using spectrophotometric methods. The medium supernatant of undegraded DR81 and the decolorized supernatants after centrifugation of suspensions at 4800× *g* were directly subjected to ultraviolet-visible (UV-vis) spectral scan analysis in the range of 250–600 modified from Rasheed et al. [38]. Fourier transform infrared (FTIR) was performed on the resolubilized methanolic fraction according to Sahasrabudhe, Saratale, Saratale, and Pathade [19].

#### 2.8.2. Hyphenated Techniques

The resolubilized methanolic fraction of degraded metabolites from the selected mixed cultures were subjected to various chromatographic techniques HPLC-UV, thin-layer chromatography coupled with mass spectroscopy (TLC-MS) and HPLC-MS for the identification of metabolites structures. HPLC-UV was performed by following the protocol of Sahasrabudhe, Saratale, Saratale, and Pathade [19] using the YL9100 HPLC system (Young In Chromass, Gyeonggi-do, Korea). A total of 10 µL volume was injected to Phenomenex ULTRACARB 7 ODS 20 C_18_ (250 × 4.6 mm) column, a gradient mobile phase was employed composed initially of 100% water, after 5 min the water:methanol mixture was (50:50), finally after 15 min, the mixture was 100% methanol at a flow rate of 0.5 mL/min. Peak detection was performed using UV-detector YL9120 (Young In Chromass, Gyeonggi-do, Korea) at ʎ_max_ 397 nm.

TLC-MS was performed using TLC silica gel 60 RP-18 F_245s_ (Merck, Darmstadt, Germany) and methanol:water (50:50), as a solvent system. Mass spectra for the desired TLC spots were measured using Advion plate express with a compact mass spectrometer (Ithaca, NY, USA), using the positive and negative electrospray ionization (ESI) polarity.

HPLC-MS protocol was performed using a reverse-phase ACQUITY UPLC–BEH C_18_ (1.7 µm–2.1 × 50 mm) column (Waters, Milford, MA, USA). The mobile phase was composed of two solvent systems, solvent A: water + 0.1% formic acid and solvent B: acetonitrile + 0.1% formic. The gradient run was set for 0–32 min/run using a flow rate of 0.2 mL min^−1^ starting initially with B 10% (2 min), B 30% (5 min), B 70% (15 min), B 90% (22 min), and finally B 100% (26 min) till the end of the run. Mass detection of peaks was achieved using Xevo TQD triple quadrupole mass spectrometer (Waters, Milford, MA, USA); the ESI^+/−^ ion acquisition mode was used. The protocol was modified from Elfarash, Mawad, Yousef, and Shoreit [20].

### 2.9. Toxicity Studies

The phytotoxic effect of the parent dye and the decolorized dye metabolites of the selected mixed cultures were evaluated on the growth of *Lepidium sativum* (water cress) seeds. Filter-sterilized (3 mL), serially diluted metabolized supernatants (1:1, 1:2, 1:4) of DR81 and decolorized metabolized supernatants were used, and the percentage phytotoxicity was calculated according to modified Hashem, Samir, Essam, Ali, and Amin [28]. The percentage germination was also calculated according to Formula (2):

(2) $$\%\;\mathrm{Germination}=\left[\frac{\;\mathrm{Number}\;\mathrm{of}\;\mathrm{germinated}\;\mathrm{seeds}}{\mathrm{Total}\;\mathrm{number}\;\mathrm{of}\;\mathrm{seeds}\;}\right]\times 100$$

The acute toxicity of the untreated dye in medium and the decolorized supernatants in dilutions 1:1, 1:2, and 1:4 in artificial seawater (ASW) was tested on aquatic life according to Ismail, Essam, Ragab, and Mourad [24]. Plain MSM-Y was diluted as the supernatant and tested to check the medium toxicity on *Artemia salina* (brine shrimp) hatched nauplii in the instarII and III stages. The number of dead nauplii was counted, and the mortality percentage was calculated according to Equation (3):

(3) $$\%\;\mathrm{Mortality}=\left[\frac{\mathrm{Number}\;\mathrm{of}\;\mathrm{dead}\;\mathrm{nauplii}}{\mathrm{Initial}\;\mathrm{number}\;\mathrm{of}\;\mathrm{live}\;\mathrm{nauplii}}\right]\times 100$$

## 3. Results

### 3.1. Determination of the Extent of DR81 Decolorization by Mixed Bacterial Cultures

**Figure 2:**
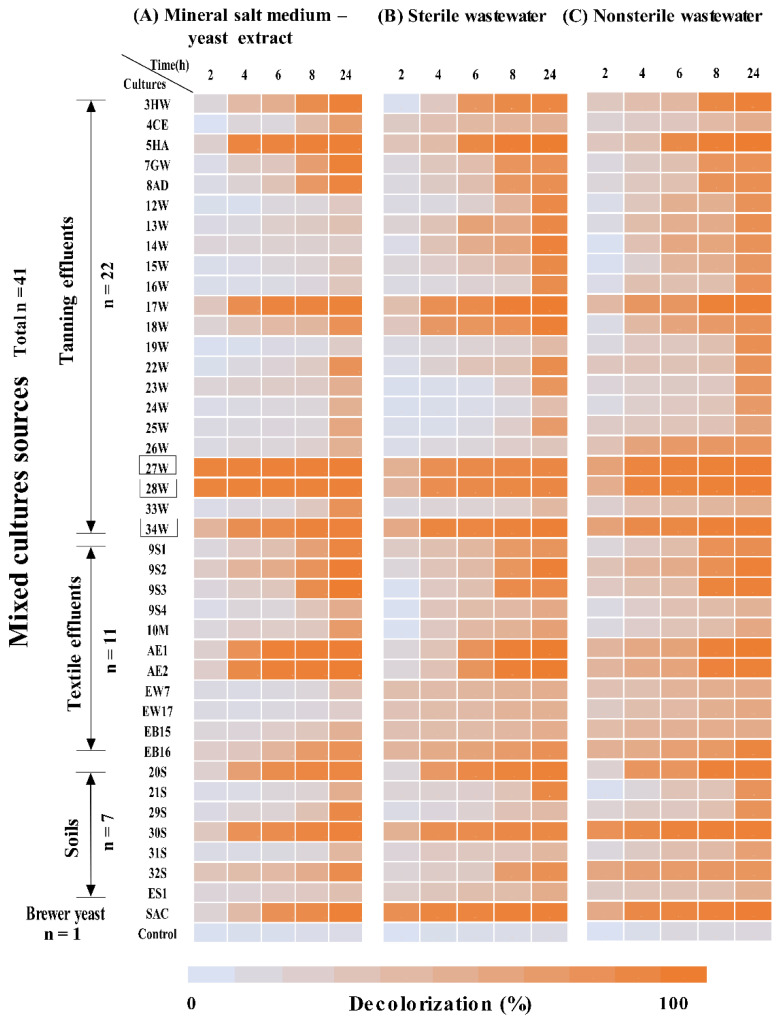
**The average extent of DR81 azo dye decolorization over time by mixed cultures in mineral salt medium and real industrial wastewater**. A heat map representing the extent of DR81 decolorization determined in (**A**) a mineral salt medium supplemented with 0.1% yeast extract, (**B**) sterile wastewater, and (**C**) nonsterile wastewater. The 3 tested media were inoculated with each of the 41 mixed cultures recovered from tanning effluents (*n* = 22), textile industrial effluents (*n* = 11), soils from contaminated sites (*n* = 7), and a brewer yeast sample. Incubation was performed at 30 °C statically, and the samples were withdrawn at 2, 4, 6, 8, and 24 h, then percentage decolorization was determined. The *X*-axis represents time points (h), and the *Y*-axis represents the labeled mixed cultures. The color code represents percentage decolorization; as the orange color intensifies it reflects higher % decolorization. Mixed cultures showing the highest decolorization (≥80% at 8 h) are highlighted in boxes.

After preliminary screening of the 67 samples, 41 mixed cultures were isolated and showed decolorization, as demonstrated in the experimental design (Figure 1). A total of 12 developed mixed bacterial cultures showed ≥80% decolorization of 40 mg/L DR81 after 8 h in MSM-Y, SWW, and NSWW, while only the 3 best-mixed cultures 27W, 28W, and 34W were selected for further testing (Figure 2). Cell pellets obtained after centrifugation were buff or creamy white with no adsorbed color (Table S3). Single isolates from the selected mixed cultures were also tested and showed no visible decolorization after 24 h (Figure S2); however, mixed cultures showed the best decolorization results (Figure 2). Moreover, the physicochemical parameters of real NSWW changed after decolorization for the three selected mixed cultures (Table S1). Chemical oxygen demand (COD) decreased by >75%, while biological oxygen demand (BOD) decreased by >90%. Further, the total nitrogen, ammonium, and nitrates decreased by 50%, 99%, and 50%, respectively. Moreover, there was >50% decrease in phenols, more than 98% decrease in bicarbonates, and an 8% decrease in total hardness.

### 3.2. Effect of Initial Dye Concentration on Decolorization in Real Industrial Wastewater

**Figure 3:**
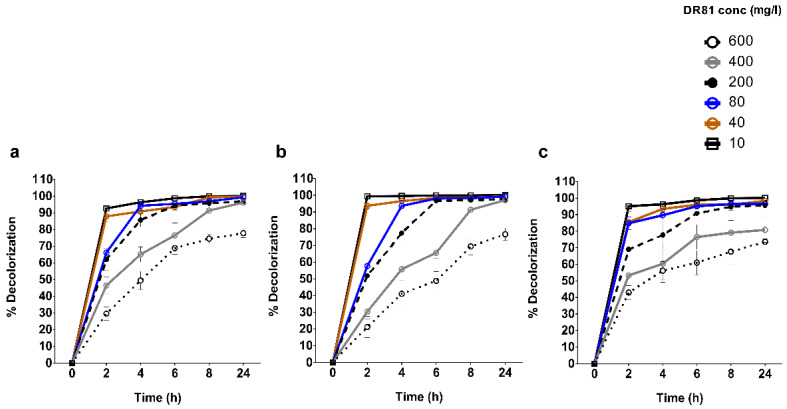
**Effect of initial dye concentration on the extent of decolorization in real industrial wastewater over time with the three selected mixed cultures.** Different concentrations of DR81(10, 40, 80, 200, 400, 600 mg/L) were prepared in non-sterile wastewater (NSWW) and inoculated each with the selected mixed cultures (**a**) 27W, (**b**) 28W, and (**c**) 34W isolated from tanning effluents. The samples were withdrawn at 2, 4, 6, 8, and 24 h, then the percentage decolorization was determined. The *X*-axis represents time points (h), and the *Y*-axis represents percentage decolorization as described in the materials and methods. The data represent at least two biological replicas, statistical analysis was performed using two-way ANOVA with Tukey’s multiple comparison test, and results are expressed as mean % decolorization ± SD.

To test the capacity of the isolated bacterial mixed cultures to decolorize DR81, the effect of increasing dye concentration (10 to 600 mg/L) was monitored in real wastewater. At concentrations 10 to 400 mg/L of DR81, the rate of decolorization was >80% at 6–24 h (Figure 3). The selected mixed bacterial cultures could tolerate up to 600 mg/L of DR81, showing >70% decolorization after 24 h (Figure 3).

### 3.3. Factors Affecting DR81 Decolorization

**Figure 4:**
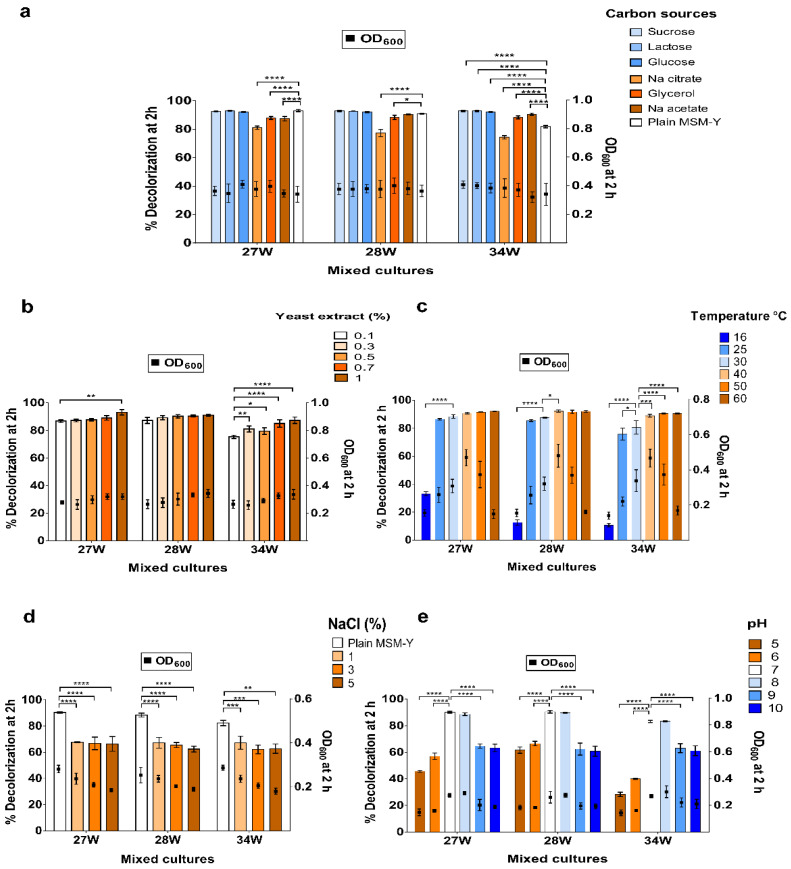
**Factors affecting DR81 decolorization at 2 h by the selected mixed cultures.** Each bar chart represents a tested factor affecting DR81 decolorization by the selected mixed cultures 27W, 28W, and 34W. The tested factors were (**a**) carbon sources, (**b**) yeast extract concentration, (**c**) temperature, (**d**) NaCl concentration, and (**e**) pH. The *X*-axis represents the selected mixed cultures labels, and the left *Y*-axis represents percentage decolorization, which was plotted as bars, while the right *Y*-axis represents OD_600_ at 2 h plotted as black squares. The data represent at least three biological replicas, and statistical analysis was performed using two-way ANOVA with Tukey’s multiple comparison test, and the results are expressed as mean % decolorization ± SD. Significance of % decolorization was defined as * *p* ≤ 0.05, ** *p* ≤ 0.01, *** *p* ≤ 0.001, **** *p* ≤ 0.0001.

Different carbon sources were tested. Glucose, glycerol, sucrose, and lactose showed >90% decolorization after 2 h while >74% decolorization with sodium citrate (Figure 4a). Increasing yeast extract from 0.1% to 1% increased the decolorization from 86% to 93% for the mixed culture 27W, while the decolorization increased from 75% to 87% for the mixed culture 34W (Figure 4b). Various incubation temperatures were tested, and it was found that the selected mixed bacterial cultures achieved >90% decolorization at 60 °C at 2 h (Figure 4c). After 2 h of incubation at various temperatures, the OD_600_ was significantly higher at 30 to 50 °C, irrespective of the ability of the mixed cultures to decolorize DR81. To test the effect of salinity, increasing concentrations of NaCl were used. After a 2 h interval, 27W, 28W, and 34W tolerated salinity up to 5%, showing >60% decolorization (Figure 4d). Interestingly, the selected mixed cultures tolerated 1% and 3% NaCl with >80% decolorization and 5% NaCl with >70% decolorization after 4 h (Figure S1b).

After 2 h, mixed cultures 27W, 28W, and 34W could tolerate pH 8 by >80% decolorization while at pH 9 and pH 10 showed >60% decolorization. In acidic conditions, the selected mixed cultures showed >40% decolorization (Figure 4e). Decolorization dramatically increased after 4 h, mixed cultures 27W and 34W showed >90% decolorization, and the mixed culture 28W showed >85% decolorization at pH 8, 9, and 10. Meanwhile, in acidic conditions, the mixed culture 28W showed >85% decolorization at pH 5 (Figure S1c).

The selected mixed bacterial cultures showed non-significant differences between various inoculum sizes with the maximum decolorization (90%) for the mixed culture 34W at inoculum 20% in 2 h. No visible decolorization was detected among shaken cultures. After 2 h, the mixed cultures 27W, 28W, and 34W showed 88.1%, 87.4%, and 73.2% decolorization under static conditions, respectively. However, under shaking conditions, decolorization at 2 h by mixed cultures 27W, 28W, and 34W dropped to 1.6%, 1.9%, and 2.7%, respectively.

### 3.4. Molecular Identification of Members of the Selected Mixed Bacterial Cultures

PCR products for *16S rRNA* gene of the culturable single colonies from the selected mixed bacterial cultures were sequenced. Multiple sequence alignment results showed that the mixed culture 27W consisted of 6 different bacteria, 28W had 5 bacteria, and 34W had 4 bacteria, as shown in Table 1. Members of mixed cultures 27W and 28W showed high similarity where gram-negative *Pseudomonas* sp. and *Stenotrophomonas maltophilia* were identified and the gram-positive *Paenibacillus agaridevorans* was common. Members of the mixed culture 34W were different from the other two mixed cultures as *Alcaligenes faecalis*, *Brevundimonas diminuta*, and *Pseudochrobactrum* sp. were identified.

**Table 1:** Identification of single isolates from the selected mixed cultures.

Mixed Cultures	Isolates	Identification	Max Score	Total Score	% Identity ^1^	Accession
27W	A	*Stenotrophomonas maltophilia*	1840	7361	99.22%	CP044092.1
	B	*Proteus vulgaris*	1812	1812	99.90%	MN685224.1
	C	*Achromobacter xylosoxidans*	1921	1921	99.90%	MN904889.1
	D	*Pseudomonas* sp. YP1	1652	1652	99.56%	KF719297.1
	E	*Pseudomonas monteilii*	1531	1531	99.76%	MN889010.1
	F	*Paenibacillus agaridevorans*	1886	1886	99.90%	KU922394.1
28W	A	*Agromyces mediolanus*	2065	2065	99.82%	MF459693.1
	B	*Stenotrophomonas maltophilia*	1927	7708	100.00%	CP044092.1
	C	*Paenibacillus agaridevorans*	1886	1886	99.90%	KU922394.1
	D	*Pseudomonas* sp. YP17	1565	1565	100.00%	KF719295.1
	E	*Pseudomonas taiwanensis*	1940	1940	99.81%	MN082103.1
34W	A	*Alcaligenes faecalis*	1869	1869	99.90%	MN515060.1
	B	*Brevundimonas diminuta*	1943	1943	99.72%	MN923411.1
	C	*Pseudochrobactrum* sp.	1592	1592	100.00%	KC337108.1

^1^ Query cover was 100%, and E-value = 0 for all identified isolates.

### 3.5. Screening for Reductive and Oxidative Degrading Enzymes

Screening for the degradative enzymatic activity for 27W, 28W, and 34W mixed cultures showed significant azoreductase activity at 2 h compared to the control cells at 0 time, showing activity of 1.39, 1.52, and 1.81 µM DR81 reduced/min/mg protein, respectively, *p* ≤ 0.0001 (Table 2). Low activity of laccase and lignin peroxidase was observed at 2 h; however, they were significantly higher than 0 time (Table 2). Similarly, low veratryl alcohol oxidase activity was observed for 27W and 28W but not 34W at 2 h (Table 2). In the mixed culture 34W, a slightly higher activity of laccase and lignin peroxidase 0.77 and 0.75 U/min/mg, respectively, was observed at 2 h. There was no significant difference in the activity of azoreductase, laccase, and lignin peroxidase between the mixed cultures. However, veratryl alcohol oxidase activity was significantly higher in 28W than 34W (Table 2).

### 3.6. Azoreductase Optimum Activity

**Figure 5:**
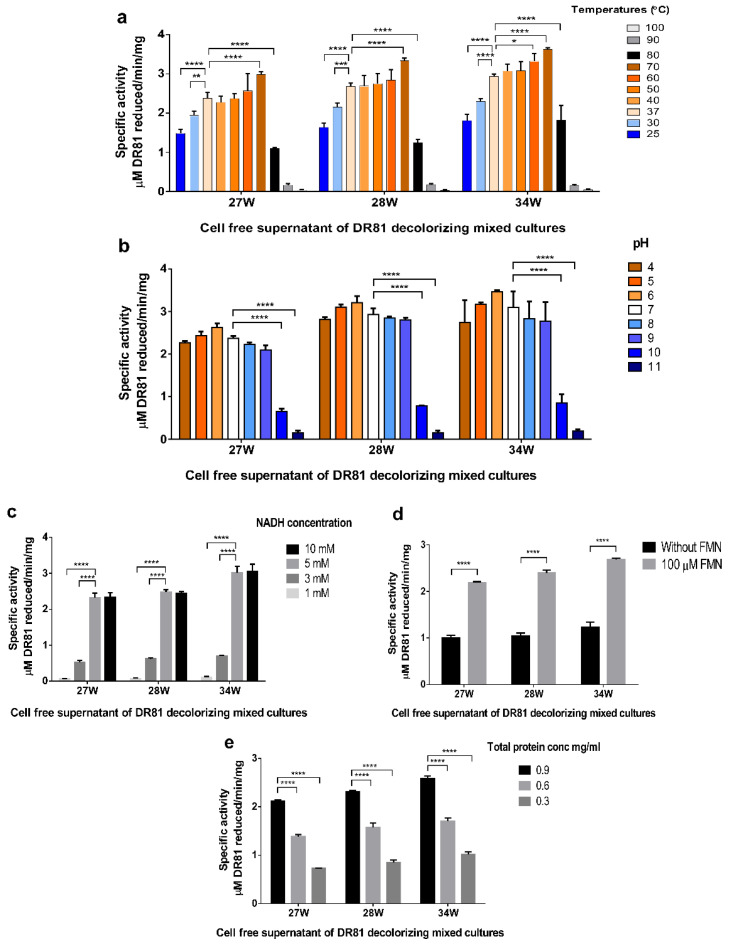
**Azoreductase activity of the crude enzyme in the selected mixed cultures.** Each bar chart represents a tested factor in obtaining optimum activity conditions (**a**) temperature, (**b**) pH, (**c**) NADH concentration, (**d**) FMN dependence, and (**e**) crude enzyme concentration. Azoreductase activity was assessed using the cell-free supernatant of the selected mixed cultures 27W, 28W, and 34W. The *X*-axis represents mixed culture labels, and the *Y*-axis represents azoreductase-specific activity as µM DR81 reduced/min/mg total protein. Optimum activity of the crude enzyme in the selected cultures was obtained at 70 °C, pH 6, 5 mM NADH and FMN dependence with thermotolerance over 25–70 °C and pH tolerance over pH 4–9. Data represent at least three biological replicas, and statistical analysis was performed using two-way ANOVA with Tukey’s multiple comparison test, and results were expressed as mean specific activity ± SD. Significance of enzymatic activity was defined as * *p* ≤ 0.05, ** *p* ≤ 0.01, *** *p* ≤ 0.001, **** *p* ≤ 0.0001.

It was noticed that upon increasing temperatures from 25 to 70 °C, there was an increase in activity, with optimum activity at 70 °C. When the temperature was further increased to 80 °C, azoreductase activity dropped, and there was little to no activity at 90 and 100 °C, respectively (Figure 5a). It was found that the selected mixed bacterial cultures remained active over a wide range of pH from 4 to 9. Azoreductase activity was observed in acidic conditions at pH 4–6, and in alkaline conditions at pH 8–9 showing a non-significant difference (Figure 5b). The optimum NADH concentration was 5 mM with no significant difference at 10 mM (Figure 5c). Moreover, it was noticed that azoreductase activity decreased to half in the absence of FMN (Figure 5d).

### 3.7. Azoreductase Stability Testing

**Figure 6:**
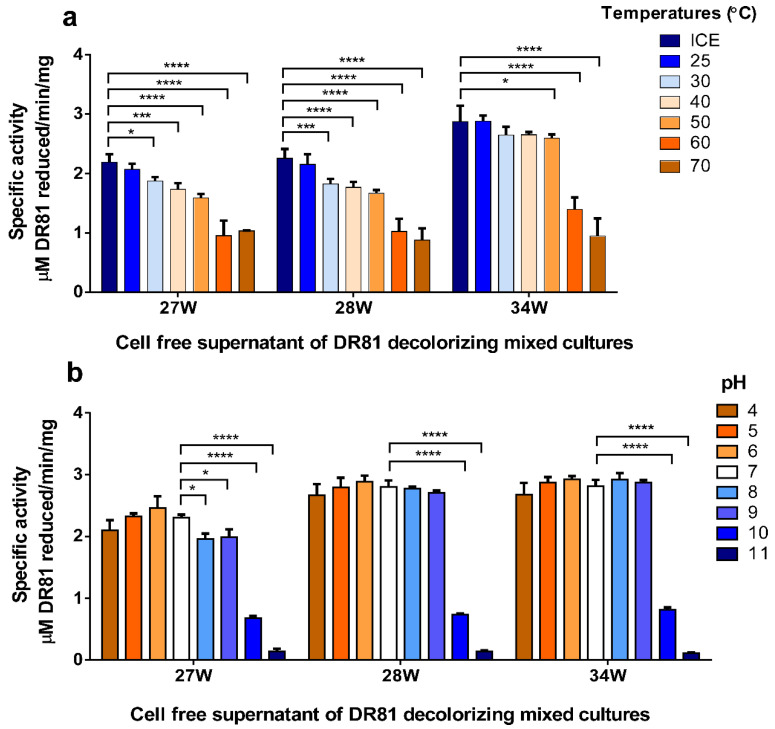
**Crude azoreductase thermal and pH stability in the selected mixed cultures.** (**a**) Crude azoreductase temperature stability, (**b**) crude azoreductase pH stability. Each bar chart represents a tested factor for crude azoreductase stability. For stability testing, the crude azoreductase activity was determined after 1 h exposure of the cell-free supernatants for the selected cultures 27W, 28W, and 34W to different temperatures or different buffers of variable pH. The *X*-axis represents mixed culture labels, and *Y*-axis represents azoreductase-specific activity as µM DR81 reduced/min/mg total protein. Data represent at least three biological replicas, and statistical analysis was performed using two-way ANOVA with Tukey’s multiple comparison test, and results were expressed as mean specific activity ± SD. Significance of enzymatic activity was defined as * *p* ≤ 0.05, *** *p* ≤ 0.001, **** *p* ≤ 0.0001.

The extracted azoreductase thermal stability was above 50 °C and a broad pH stability range from 4 to 9 (Figure 6). Azoreductase activity in the crude enzyme was stable over a wide range of temperatures after one hour of exposure (Figure 6a). Compared to the control experiment, the thermal stability of azoreductase crude enzyme was almost the same at 25 °C. Upon increasing temperatures from 30 to 50 °C, the azoreductase stability slightly decreased, while a further increase in temperature to 60 and 70 °C decreased the azoreductase stability dramatically (Figure 6a). Compared to the pH stability of the control experiment at pH 7, the crude enzymes from the selected mixed cultures showed a wide pH stability range from pH 4 to 9. A further increase of pH to 10 and 11 dramatically decreased azoreductase activity (Figure 6b).

### 3.8. Identification of Azoreductase Genes in the Selected Mixed Bacterial Cultures

**Figure 7:**
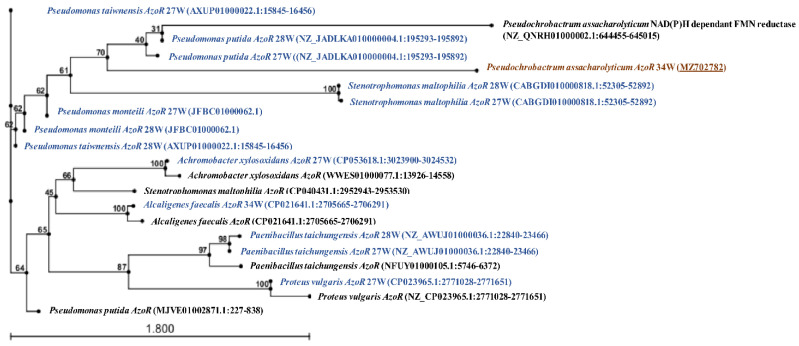
**Phylogenetic tree of the identified azoreductase genes in the selected mixed cultures.** Phylogenetic tree of *AzoR* genes identified by sequencing of their PCR products. The phylogenetic tree was generated using the nucleotide sequences of azoreductase genes detected by PCR relative to reference azoreductases by the neighbor-joining algorithm as detailed in the materials and methods section. Bootstrap values from 100 replicates are written in numbers on branches. The tree was generated using CLC main workbench 5.5. Sequences within the three mixed cultures were written in blue with an indication of the type of azoreductase enzyme. The accession number of the deposited nucleotide sequence for this study possible novel azoreductase in *Pseudochrobactrum* sp. (MZ702782) is underlined.

The possible azoreductase genes responsible for the enzymatic activities were detected by sequencing the PCR products for the *AzoR* gene. Our preliminary results suggested the presence of multiple azoreductase genes in each mixed culture that might be responsible for azoreductase activity (Figure 7). The phylogenetic tree shows homology between azoreductases from 27W and 28W from *Pseudomonas* and *Stenotrophomonas* sp. The mixed culture 34W showed homology between azoreductase from *Alcaligenes* with *Achromobacter* from the mixed culture 27W. Moreover, the mixed culture 34W showed a possible new member of azoreductase with 89% identity with FMN-dependent azoreductase gene from *Pseudochrobactrum* (Figure 7). The nucleotide sequence of the possible new member of azoreductase from the mixed culture 34W was deposited into GenBank with accession number MZ702782.

### 3.9. Extraction of Metabolites and Prediction of Biodegradative Pathway

**Figure 8:**
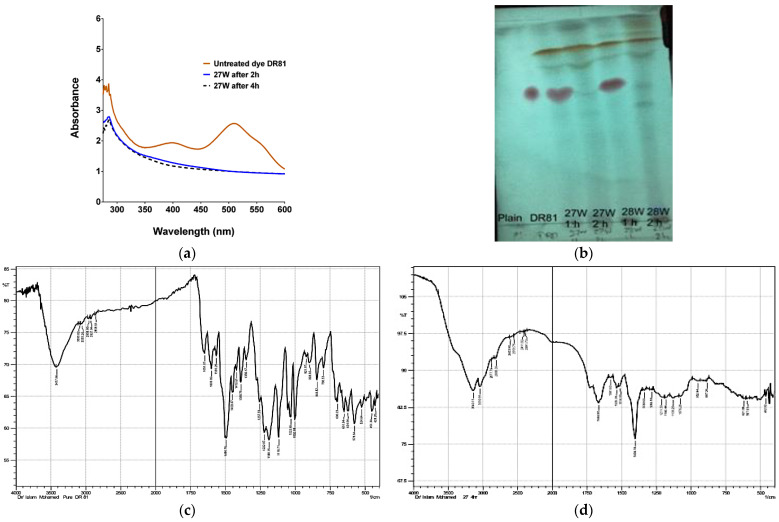

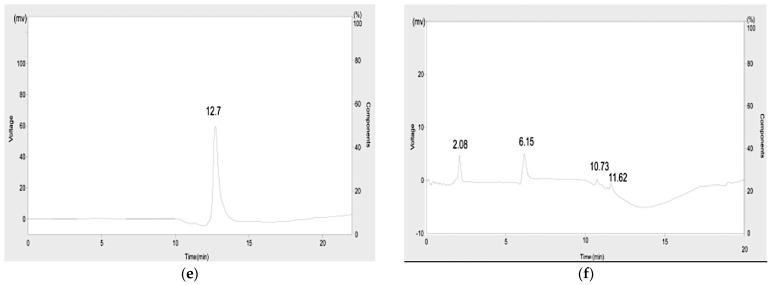
**Detection of DR81 degradation products using spectrophotometric and chromatographic methods.** (**a**) Representative UV-vis scan on the culture supernatant of mixed culture 27W at 2 and 4 h compared to 0 time undegraded supernatant. The *X*-axis represents wavelengths (nm), and the *Y*-axis represents absorbances. (**b**) Representative TLC picture visualized under UV-light for the resolubilized methanolic extracts of 27W and 28W for the plain medium, DR81, and extracted metabolites at times 1 and 2 h. (**c**,**d**) Representative FTIR spectra for the resolubilized methanolic extract for DR81 and 27W mixed culture, respectively. The *X*-axis represents frequency (cm^−1^), and the *Y*-axis represents transmittance (%). (**e**,**f**) Representative HPLC/UV chromatograms for the resolubilized methanolic extract of DR81 and 27W, respectively. The *X*-axis represents time (min), the left *Y*-axis represents voltage (mV), and the right *Y*-axis represents components (%).

UV-vis spectral analysis of culture supernatant revealed the disappearance of DR81’s major peak in the visible region at ʎ_max_ (511 nm and a decrease of the ʎ_max_ 390 nm peak in the UV region (Figure 8a). FTIR analysis of the resolubilized methanolic extract was further scanned from 400 to 4000 cm^−1^ (mid IR region). The undegraded DR81 showed the characteristic –N=N– stretching azo groups at 1562 and 1600 cm^−1^ (Figure 8c,d). The observed band at 1654 cm^−1^ indicated the –C=N– stretching and –NH bending of aromatic amine in DR81. The characteristic bands of aromatic rings in the DR81 dye molecule were evident at 848, 793, 775, 709, and 651 and 624 cm^−1^. The –SO_3_ group substituted at the meta position showed a band at 1388 cm^−1^. After complete decolorization at 4 h, there was a sharp decrease of most bands and disappearance of other bands between 1500 and 1600 cm^−1^. There was a disappearance of the 1388 cm^−1^ band for the –SO_3_ group substituted at the meta position, a decrease in intensity of characteristic bands of the aromatic ring between 650 and 800 cm^−1^, and the appearance of new bands in the aliphatic region 2877, 2808 in mixed cultures 27W and 34W.

HPLC-UV analysis of the parent dye DR81 and metabolites in the resolubilized methanolic extract at different time intervals revealed a peak for the parent dye at retention time (R_t_) 12.7 min. There was a disappearance of the DR81 peak at R_t_ 12.7 and the appearance of new peaks at R_t_ 2.08, 6.15, 10.73, 11.62 min for 27W, R_t_ 5.7, 11.73, 17.32 for 28W, and R_t_ 4.2, 11.13, 16 min for 34W (Figure 8e,f). Likewise, the TLC-MS analysis results showed one spot for the dye at retention factor (R_f_) 0.52 with *m/z* 314.6 and *m/z* 652.6 and multiple spots at different R_f_ values 0.8 with *m/z* 276 and 0.88 in the ESI^-^ negative mode (Figure 8b).

HPLC-MS was further performed for the dye DR81 and the resolubilized methanolic extracts of the selected mixed cultures at time intervals (0, 1, 2, and 4 h). At 0 time, HPLC-MS analysis revealed the presence of the parent dye DR81 at R_t_ 26.06 min with *m/z* 314.7 and *m/z* 652.7 in the ESI^-^ negative mode (Figure S3a). After 1 h, a metabolite appeared in the three mixed cultures at R_t_ 13.5 min with *m/z* 276 and 278 in negative and positive ions, respectively, assigned as ((E)-4-((4-aminophenyl)diazenyl)benzenesulfonic acid) (Figure S3b). After 2 h, 3 metabolites appeared at R_t_ 25.9, R_t_ 0.79, and R_t_ 1.15 min showing *m/z* 383, *m/z* 166, and *m/z* 144, respectively (Figure S3c,d). Moreover, a metabolite appeared after 1 h at R_t_ 1.8 min ESI^+^ showed *m/z* 110 assigned as (benzene-1,4-diamine) (Figure S3e). Another metabolite was at R_t_ 13.5 min with *m/z* 371 ESI^-^ (sodium 7-benzamido-3-diazenyl-4-hydroxynaphthalene-2-sulfonate) appeared after 1 h and continued to appear till 4 h (Figure S4a). After 4 h, metabolite *m/z* 371 was detected. Moreover, there was a metabolite with *m/z* 293 ESI^-^ after 1 h at R_t_ 20.6 min assigned as N-(6-hydrazinyl-5-hydroxynaphthalen-2-yl)benzamide (Figure S4b) and another metabolite with *m/z* 167 appeared after 1 h at R_t_ 1.2 min assigned as naphthalen-1(8aH)-one and both metabolites continued to appear till 2 h (Figure S4c).

### 3.10. Toxicity Studies

The percentage germination of *Lepedium sativum* from decolourized culture supernatants was (100%) when compared to DR81 dye (64.5%) at 1:4 dilution. Percentage phytotoxicity of the selected mixed cultures 27W, 28W, and 34W was 4.37%, 4.26%, and 3.95%, respectively, while DR81 dye showed high phytotoxicity (67.5%). Moreover, acute toxicity was assessed using *Artemia salina* lethality test, where 1:1 dilution of metabolized supernatants to ASW showed 100% mortality while the plain medium showed 80% mortality. However, at 1:2 dilution of metabolized supernatants to ASW, 0% mortality was observed for all metabolized supernatants and plain diluted MSM-Y medium compared to DR81 (66%).

## 4. Discussion

The development of cost-effective and ecofriendly biodegradative microorganisms for azo dyes degradation is demanding. In the current study, preliminary screening of DR81 decolorizing mixed bacterial cultures showed that the best decolorization was obtained in the presence of 0.1% yeast extract as it contained essential cofactors for azo bond reduction such as riboflavin to perform the enzymatic reaction [39]. Moreover, it has been reported that azo dye degradation through azo bond reduction under anoxic conditions requires cofactors, such as NADH or FAD, as they serve as electron donors in the cell [3,40]. Therefore, in the current study, upon increasing yeast extract concentrations to 1%, the decolorization increased within 2 h, in agreement with previous studies [2,28]. No visible decolorization was detected among single isolates from mixed cultures (Figure S2), as mixed cultures are known to be superior due to consumption of the produced toxic metabolites by other microorganisms present in the mixed culture [10,16,41]. In the current study, the extent of decolorization of the screened mixed cultures was performed in MSM-Y along with sterile and non-sterile real wastewater to test decolorization under real conditions in the industry. The three best decolorizing mixed cultures, labeled 27W, 28W, and 34W, were selected on the bases of achieving ≥80% decolorization within 8 h incubation. The decolorization capability of the chosen mixed bacterial cultures was suggested to be due to biodegradation but not adsorption, as the effect of adsorption was excluded by observing the cell pellets, which were buff or creamy white after centrifugation of the decolorized suspensions (Table S3). It was previously reported that decolorization by adsorption to the cell wall is observed to occur more in fungi than bacteria [2,14]. 

Real effluents may contain varying amounts of suspended solids and different pH, usually effluents are high in temperature, with high COD, salt concentrations, and heavy metals due to the used chemicals during textile dyeing or tanning processes. All of these factors could be inhibitory to microbes used for biodegradation [19,42,43]. In the current study, it was shown that the composition of NSWW changed after decolorization by the selected mixed cultures suggesting that decolorization was due to biodegradation (Table S1). As the COD and BOD are considered important parameters to evaluate the degree of pollution, their decrease represents biodegradation. In this study, it was found that the untreated effluent containing high COD and TDS might be due to organic matter and salts, while high BOD suggested low dissolved oxygen due to organic matter, as previously described by Saxena, Purchase, Mulla, and Bharagava [10] and Saxena et al. (2020). In this study, the COD decreased by >75%, while the BOD decreased by >90% after decolorization, which suggested biodegradation. Moreover, our results showed that there was efficient removal of phosphates, nitrates, ammonium, total nitrogen, phenols, and bicarbonates and a decrease in total hardness, which might be due to the biotransformation of those chemicals or utilization as nutrients by the mixed cultures [10].

In addition, upon determination of the effect of initial dye concentration on decolorization, non-sterile wastewater was used to mimic the real conditions in industrial effluent. The highest decolorization capacity obtained was >70% of 600 mg/L DR81 concentration at 24 h. Increasing the initial dye concentration affects dye decolorization with multiple possible factors [19]. This decolorization inhibition might be due to the toxic effect of the dye on bacterial cells or due to the blockade of active sites on decolorizing enzymes with a lot of dye molecules. Moreover, it was reported that sulphonated azo dyes, such as our used DR81, cause inhibition of microbial growth, leading to decreased ability for decolorization [16,44]. According to Sahasrabudhe, Saratale, Saratale, and Pathade [19], greater than 80% DR81 decolorization was observed at 500 mg/L, while lower decolorization was noticed at 600–700 mg/L. In the current study, it was observed that the decolorization of DR81 by the selected bacterial mixed cultures increased when an external carbon source was added, such as glucose, glycerol, sucrose, or lactose. This might be due to the enhancement of bacterial growth and transfer of reducing equivalents to the dye to achieve azo bond cleavage [9]. Further, in this study, it was observed that decolorization decreased with sodium citrate; this might be due to the preference of bacteria to assimilate citrate over the dye as a carbon source [42].

One of the challenges of wastewater treatment is the exposure to high environmental temperatures making thermostable biodegrading bacteria important for industrial applications. For example, in textile industries, it is preferable that the biodegrading bacteria operate at high temperatures of up to 60 °C [7]. Moreover, incubation temperature affects bacterial growth, dye solubility, enzymatic activity, and reaction rate [45]. In this study, the high decolorization ability that was observed at 60 °C might be attributed to the retainment of the enzymatic activity at relatively low growth (OD_600_ = 0.15–0.17). Another challenge of azo dye-contaminated effluent is that wastewater from textile processing and dyestuff contains various alkalis, acids, and salts that are used in the brine wash step and also used to separate organic contaminants to help precipitation of the dyestuff [46]. Thus, a salt-tolerant microorganism capable of decolorizing and detoxifying azo dyes is required for industrial application. In the current study, the selected mixed cultures showed 60% decolorization in high salinity (5% NaCl) at 2 h, and with increasing time (4 h), the salinity tolerance reached >70% decolorization. In plain media, these mixed cultures showed 80% decolorization within 2 h; this reduction in decolorization to 60–70% in 5% NaCl is expected to be due to inhibition of bacterial activities at NaCl concentrations above 1% [47].

Another important factor considered in choosing effective decolorizing agents is tolerating a wide range of pH, as during the textile and tanning industrial processes, the color and solubility of some dyes depend on pH. This study showed a wide range of pH tolerance profiles as the mixed cultures 27W, 28W, and 34W tolerated alkali conditions with better decolorization from pH 7 to 10. According to Solís, Solís, Pérez, Manjarrez, and Flores [9], bacterial decolorization was optimal at pH 6 to 10, while a preference for alkaline conditions was observed by Agrawal et al. [48]. As for increasing the inoculum size, it had no effect on decolorization; this could be considered an advantage over other studies that showed reduced degradation upon a decrease in the inoculum size [49,50]. In addition, decolorization was only observed in static cultures, as it might be due to the reductive pathway of azoreductase rather than the oxidative pathway [16].

Screening for reductive and oxidative enzymatic activity for the three selected mixed cultures showed the induction of strong azoreductase activity; this might be related to the observed lack of decolorization with agitation. Moreover, traces of other oxidative enzymes, namely laccase, veratryl alcohol oxidase, and lignin peroxidase, were observed in the current study, which might be responsible for synergism with azoreductase towards degradation of azo dye with non-toxic metabolites [51,52]. Another possible reason for synergistic enzymatic degradative ability is the possible presence of other undetermined oxidative enzymes that might play a role in a multi-enzymatic pathway towards DR81 biodegradation [53]. Since azoreductase was predicted to be the major enzyme responsible for decolorization, optimum conditions for enzymatic activity were studied in the selected mixed cultures. Azoreductases are classified as flavin-dependent and flavin independent [16]. Hence, cofactor requirement such as NADH or NADPH by azoreductase is very important as they serve as electron donors to azo bond for cleavage [54]. In this study, the crude enzyme in the three mixed cultures exhibited optimum activity at 70 °C, pH 6, 5 mM NADH, and FMN dependance with thermotolerance over 25–70 °C and pH tolerance over pH 4–9. The decrease in azoreductase activity in the absence of FMN suggested that crude azoreductazes were FMN-dependent. Azoreductase with a broad optimum temperature of 65 to 80 °C was found by Ooi, Shibata, Sato, Ohno, Kinoshita, Thuoc, and Taguchi [34], with temperature stability below 45 °C and more than half of activity at 65 °C and broad pH stability profile after 1 h exposure to pH 6–10. As the gene and protein sequences of azoreductases share a low-level sequence similarity among different bacteria, we designed primers for each identified bacterium based on our molecular identification of bacteria within each mixed culture to the species level [46]. Some of these designed primers were degenerated to allow better identification of any possible novel enzyme and were designed according to the azoreductase database by Zahran et al. [55]. In the current study, molecular identification and sequencing of azoreductase-coding genes from the selected mixed cultures showed the presence of multiple azoreductase genes in each mixed culture, which might play different roles in azo dye biodegradation as previously reported in the literature [56]. Moreover, the homology between azoreductases detected in the mixed cultures 27W and 28W and some azoreductases from 34W suggested the close patterns of biodegradation as represented by the phylogenetic tree. The mixed culture 34W showed a possible new member of azoreductase with 89% identity with FMN-dependent azoreductase gene from *Pseudochrobactrum*. This bacterium might have played a role in the decolorization of DR81 azodye, as described earlier by Siddique et al. [57], who demonstrated the degradation of different dyes using biogenic nanoparticles of *Pseudochrobactrum* sp. C5.

In the current study, to identify the nature of the biodegradation products derived from DR81 dye, several chromatographic, spectrometric, and chromatographic techniques coupled with spectrophotometric techniques were employed. It was found that in the UV-vis spectral scan for decolorized supernatants, the disappearance of the major peak of DR81 at ʎ_max_ 511 nm indicated decolorization similar to that described by Al-Shareef et al. [58], while the decrease in ʎ_max_ 390 nm peak in the UV-region indicated degradation. Moreover, in the FTIR spectrometry for the resolubilized methanolic extract, after complete decolorization at 4 h, the sharp decrease of most bands and disappearance of other bands between 1500 and 1600 cm^−1^ was strong evidence of azo bond cleavage and degradation, as reported by Amin, Rastogi, Chaubey, Jain, Divecha, Desai, and Madamwar [21]. Moreover, the disappearance of the 1388 cm^−1^ band for –SO_3_ group indicated desulfonation, while the appearance of new bands in the aliphatic regions 2877 and 2808 in the mixed cultures 27W and 34W and the decrease in the intensity of some characteristic bands of the aromatic ring between 650 and 800 cm^−1^ suggested degradation and ring cleavage. Similar results were observed by Amin, Rastogi, Chaubey, Jain, Divecha, Desai, and Madamwar [21] for the degradation of DR81 by *Bacillus* sp. DMS2 and Rathour et al. [59] for the degradation of raw textile effluent by bacterial community DR4. HPLC-UV and TLC-MS provided confirmatory results of DR81 degradation in the resolubilized methanolic extract. The disappearance of the major peak for the parent dye and the appearance of new peaks for the extracted metabolites at different R_t_ in HPLC-UV, as well as the formation of new spots in the TLC with different R_f_ indicated degradation. Similar results were obtained by Ramadan et al. [60] and Sahasrabudhe, Saratale, Saratale, and Pathade [19].

**Figure 9:**
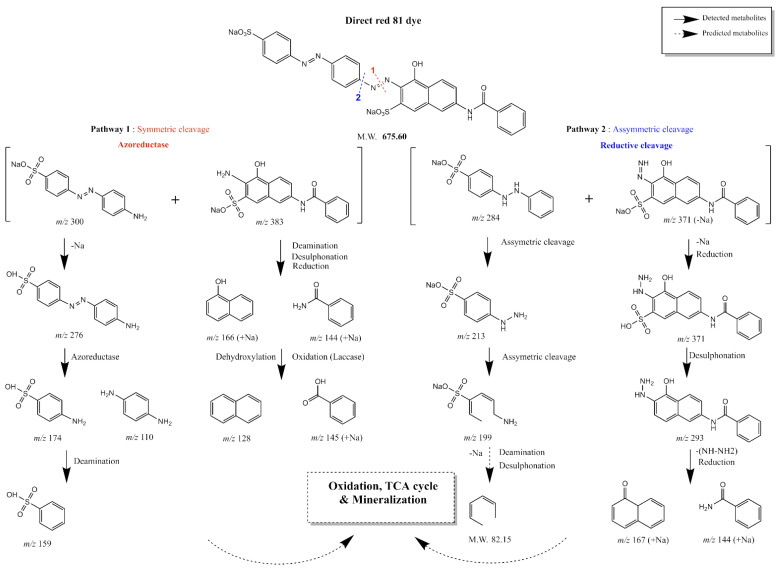
**Proposed pathway for the degradation of DR81 by mixed cultures 27W, 28W, and 34W based on detected masses from TLC-MS and HPLC-MS.** The proposed pathway suggested DR81 enzymatic cleavage through two reductive pathways based on the metabolites detected by TLC-MS and HPLC-MS. The bond cleavage of the DR81 structure was represented by colored and numbered dotted lines on the parent dye chemical structure, where the red dotted line (1) demonstrates symmetric cleavage by azoreductase, while the blue dotted line (2) demonstrates asymmetric reductive cleavage. The black solid arrows represent structure degradation to a detected metabolite by TLC-MS or HPLC-MS where *m/z* was written down for each detected metabolite. The black dotted arrows represent structure degradation to a predicted metabolite. The pathway was generated by ChemDraw Professional 15.

To more specifically identify degradative metabolites chemical structure to aid in determining the involved biodegradative pathways, TLC-MS and HPLC-MS were performed on the DR81 dye and the resolubilized methanolic extracts of the selected mixed cultures at time intervals (0, 1, 2, and 4 h). At zero time, HPLC-MS analysis confirmed TLC-MS results revealing the presence of the parent dye, DR81 (Figure S3a) [61]. Mass analysis for the extracted metabolites showed two biodegradative pathways, symmetric cleavage of the azo bond (pathway 1) and asymmetric cleavage (pathway 2) (Figure 9). After 1 h, the major intermediate metabolite by azoreductase in the 3 mixed cultures was at R_t_ 13.5 min with *m/z* 276 and 278 in negative and positive ions, respectively, assigned as (E)-4-((4-aminophenyl)diazenyl)benzenesulfonic acid (Figure S3b), which was in accordance with the mass detected in the TLC major spot at R_f_ 0.8 (Figure 8b). Such a pattern of detected metabolite peaks showed that the initial step of degradation was due to the symmetric reduction of the azo bond in DR81 by the azoreductase enzyme in pathway 1 (Figure 9). After 2 h, another reductive metabolite by symmetric cleavage was at R_t_ 25.9 min with *m/z* 383 assigned as sodium 3-amino-7-benzamido-4-hydroxynaphthalene-2-sulfonate, Figure S3c, which further degraded to yield *m/z* 166 (naphthalen-1-ol) and 144 (benzamide), Figure S3d, at R_t_ 0.79 and 1.15 min, respectively. 

Another metabolite formed by asymmetric cleavage in pathway 2 at R_t_ 13.5 min with *m/z* 371 ESI^−^ (sodium 7-benzamido-3-diazenyl-4-hydroxynaphthalene-2-sulfonate) appeared after 1 h and continued to appear till 4 h, Figure S4a. After 4 h, the intermediates formed by azoreductase *m/z* 276 and *m/z* 383 completely disappeared, whereas metabolite *m/z* 371, made by asymmetric cleavage, remained. Moreover, two other metabolites suggested the asymmetric reductive cleavage appeared after 1 h and continued to appear till 2 h showing *m/z* 293 and 167, respectively (Figure S4b,c). After the reduction step, traces of oxidative enzymes, such as laccase, veratryl alcohol oxidase, and lignin peroxidases, Table 2, might have caused oxidation, aromatic ring cleavage, and possible mineralization. The degradation patterns proposed that DR81 was first subjected to sequential reduction of the two azo bonds present in the structure by azoreductase and another undetermined reductive enzyme, then further oxidation, possibly by laccase, peroxidase, and veratryl alcohol oxidase or other undetermined oxidative enzymes, and the proposed pathway was elucidated, Figure 9. Similar cleavage patterns were obtained by Thakur et al. [62] for the degradation of azo dye red HE7B using *Bacillus* sp., which involved the reductive cleavage of both N=N and C-N bonds adjacent to the azo bond.

After decolorization, the possible release of toxic aromatic amines may cause adverse environmental effects. Therefore, toxicity tests are important to determine the suitability of the treated water for release into the water system with possible agricultural applications [63,64]. In this study, phytotoxicity was assessed using cress (*Lepedium sativum*), which has the advantage of rapid germination and sensitivity to low concentrations of phytotoxic chemicals, as previously mentioned by Cassano et al. [65]. In this study, serial dilutions of the CFS 1:1, 1:2, and 1:4 were used for the germination of *Lepedium sativum* compared to tap water, and the results showed that both shoot and root lengths increased at various dilutions of CFS. This indicated the possible toxicity of the plain MSM-Y medium. Moreover, in acute toxicity assay, supernatants’ dilution in ASW resulted in decreased toxicity at 1:2 dilution, which might be due to the toxic effect of the MSM-Y medium components on *Artemia salina* nauplii as the plain medium showed 80% mortality, as previously reported by Ismail, Essam, Ragab, and Mourad [24]. The decrease in water cress phytotoxicity and mortality of brine shrimp suggested detoxification of the dye and its metabolites with the production of less-toxic products [66].

## 5. Conclusions

In conclusion, this study identified and characterized three mixed-bacterial cultures that showed biodegradation of diazo dye DR81 with the ability to tolerate high dye concentrations. The selected mixed cultures tolerated harsh conditions of temperature, salinity, and pH with the capacity to decolorize and detoxify DR81 within a short time (2 h). The enzymatic activity assays along with UV-Vis, FTIR, TLC-MS, and HPLC-MS of the extracted metabolites confirmed the biodegradation of DR81. The major enzymatic activity and metabolic pathway might be attributed mainly to azoreductase activity in synergism with laccase, lignin peroxidase, and veratryl alcohol oxidase or other oxidative enzymes. The detected azoreductase from the identified bacterial member Pseudochrobactrum of the mixed culture 34W suggested a possible novel azoreductase. The biodegraded metabolites were non-toxic in plant and microanimal toxicity models. This study demonstrated mixed bacterial cultures that would be useful agents for detoxifying DR81, which can be safely implemented in industrial biodegradative applications. Industrial application of the study results should prove useful in cost and time-efficient biological treatment of complex tanning and textile effluents that have high loads of organic matter, high temperature, salinity, and pH. Possible future use of mixtures of the three mixed cultures for the treatment of industrial wastewater in situ on small scale bioreactors followed by scaling up to large scale bioreactors then real effluent treatment tanks by putting together 27W, 28W, and 34W cultures would be useful to investigate for industrial applications [67]. Future studies investigating the gene expression of the mixed cultures based on transcriptomics techniques should prove useful [68].

## Figures and Tables

**Table 2:** Oxidative and reductive enzymatic activity by crude enzymes of selected mixed cultures.

Enzymes	Time (h)	27W	28W	34W
Azoreductase ^1^	0	0.056 ± 0.027	0.026 ± 0.008	0.056 ± 0.032
	2	1.39 ± 0.30 ****^3^	1.52 ± 0.42 ****	1.81 ± 0.40 ****
Laccase ^2^	0	0.041 ± 0.049	0.063 ± 0.046	0.066 ± 0.014
	2	0.52 ± 0.13 *	0.55 ± 0.12 *	0.77 ± 0.21 ***
Lignin peroxidase ^2^	0	0.067 ± 0.038	0.087 ± 0.003	0.055 ± 0.048
	2	0.75 ± 0.32 ***	0.79 ± 0.14 ***	0.75 ± 0.30 ***
Veratryl alcohol oxidase ^2^	0	0.025 ± 0.029	0.064 ± 0.083	0.047 ± 0.063
	2	0.71 ± 0.34 ***	0.76 ± 0.34 ***^4^	0.31 ± 0.13 ^4^

^1^ Azoreductase-specific activity µM DR81 reduced/min/mg protein, ^2^ Enzymatic activity U/min/mg protein, ^3^ Significance of enzymatic activity at 2 h compared to the initial activity at 0 time was defined as * *p* ≤ 0.05, *** *p* ≤ 0.001, **** *p* ≤ 0.0001, ^4^ Veratryl alcohol oxidase activity was significantly higher than 34W. Data represent at least three biological replicates. Data analysis was performed by GraphPad prism using two-way ANOVA.

## Data Availability

The data presented in this study are available in the article and supplementary material files. The nucleotide sequence for the possible novel azoreductase in *Pseudochrobactrum* sp. was deposited to GenBank with accession number BankIt2487241 Pseudochrobactrum MZ702782.

## Supplementary Materials

The following supporting information can be downloaded at: https://www.mdpi.com/article/10.3390/microorganisms10050994/s1, Figure S1: A panel showing the DR81 calibration curve and the effect of both salinity and pH on DR81 decolorization. Figure S2: Decolorization of DR81 by single colonies isolated from the selected mixed bacterial cultures. Figure S3: Mass spectra, chemical structures, and IUPAC names for the parent dye DR81 and the possible detected metabolites in the resolubilized methanolic extract of culture supernatant from symmetric cleavage by azoreductase extracted after 1 and 2 h incubation, analyzed using HPLC-MS. Figure S4: Mass spectra, chemical structures, and IUPAC names for the possible detected metabolites in the resolubilized methanolic extract of culture supernatant from asymmetric reductive cleavage extracted after 1 and 2 h incubation, analyzed using HPLC-MS. Table S1: Physicochemical parameters of non-sterile wastewater (NSWW). Table S2: Primers used for PCR and sequencing of azoreductase genes in the selected mixed cultures. Table S3: Decolorization of DR81 azo dye by selected bacterial mixed cultures showing both decolorized suspensions and cell pellets.
