# Supplementary material for: Biodegradation, Decolorization, and Detoxification of Di-Azo Dye Direct Red 81 by Halotolerant, Alkali-Thermo-Tolerant Bacterial Mixed Cultures
Source: Microorganisms. 2022 May 9;10(5):994. doi: 10.3390/microorganisms10050994 (PMC9147255; doi:10.3390/microorganisms10050994)
Supplement: Supplementary file 1 [file microorganisms-10-00994-s001.zip › microorganisms-1705829-supplementary.pdf]

Supplementary Materials

Supplementary Figures

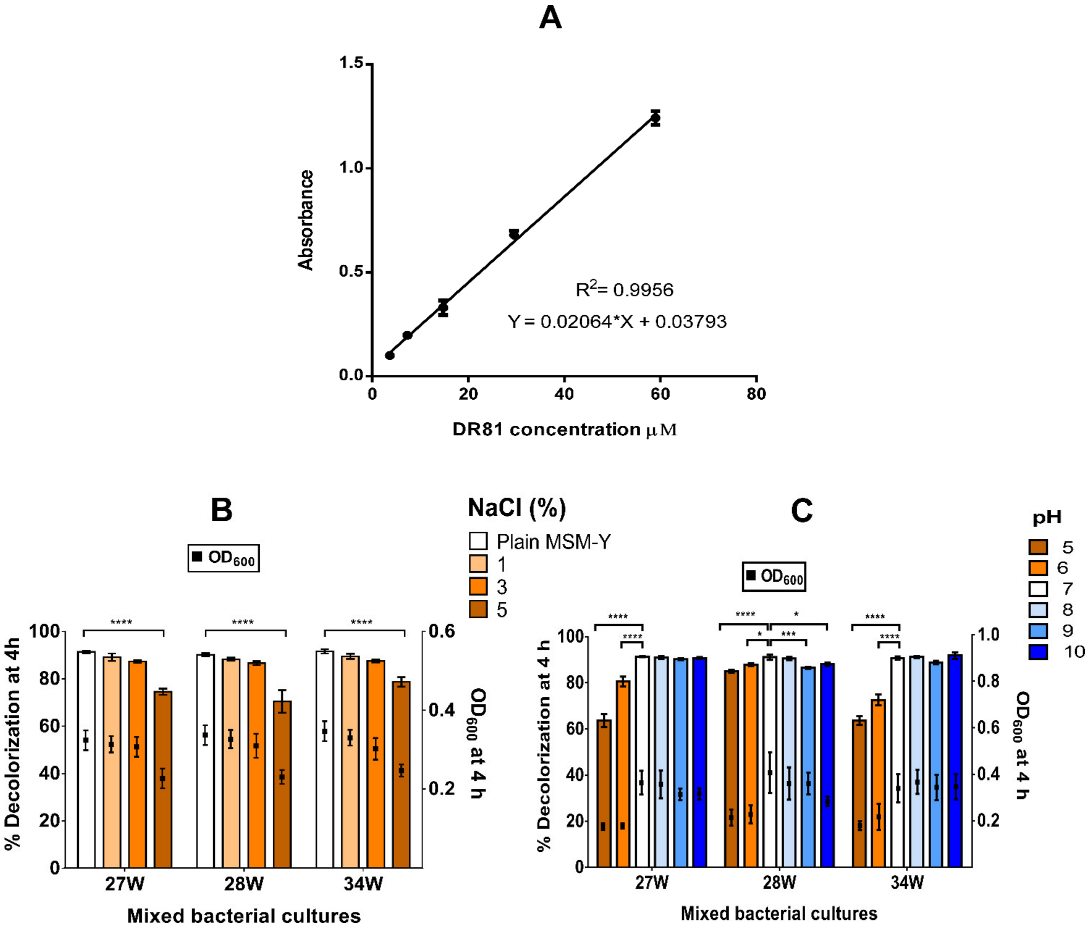

Figure S1: A panel showing DR81 calibration curve and the effect of both salinity and pH on DR81 decolorization. (**A**) Calibration curve for DR81 azo dye. Absorbances were recorded for three separate replicates and results were expressed as mean absorbance ± SD, (**B**) Effect of NaCl concentration on DR81 decolorization after 4 h by the selected mixed cultures, (**C**) Effect of pH on DR81 decolorization after 4 h by the selected mixed cultures. Data represent three replicates and analyzed by GraphPad prism using two-way ANOVA with Tukey's multiple comparison test and results were expressed as mean % decolorization ± SD. Significance of % decolorization was defined as * $p \leq 0.05$, ** $p \leq 0.01$, *** $p \leq 0.001$, **** $p \leq 0.0001$.

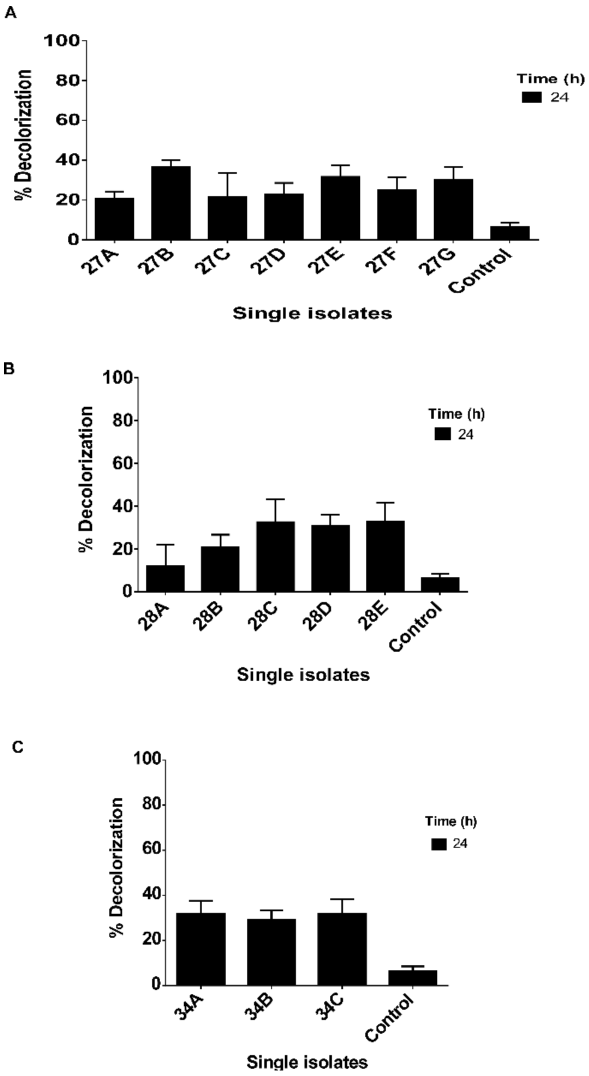

Figure S2. Decolorization of DR81 by single colonies isolated from the selected mixed bacterial cultures (**A**) 27W, (**B**) 28W and (**C**) 34W after 24 h. Single isolates showed no decolorization at time intervals 2, 4, 6 and 8 h and no visible complete decolorization was detected at 24 h when compared to their corresponding mixed culture. Data represent at least three replicates and plotted as mean % decolorization ± SD using GraphPad prism.

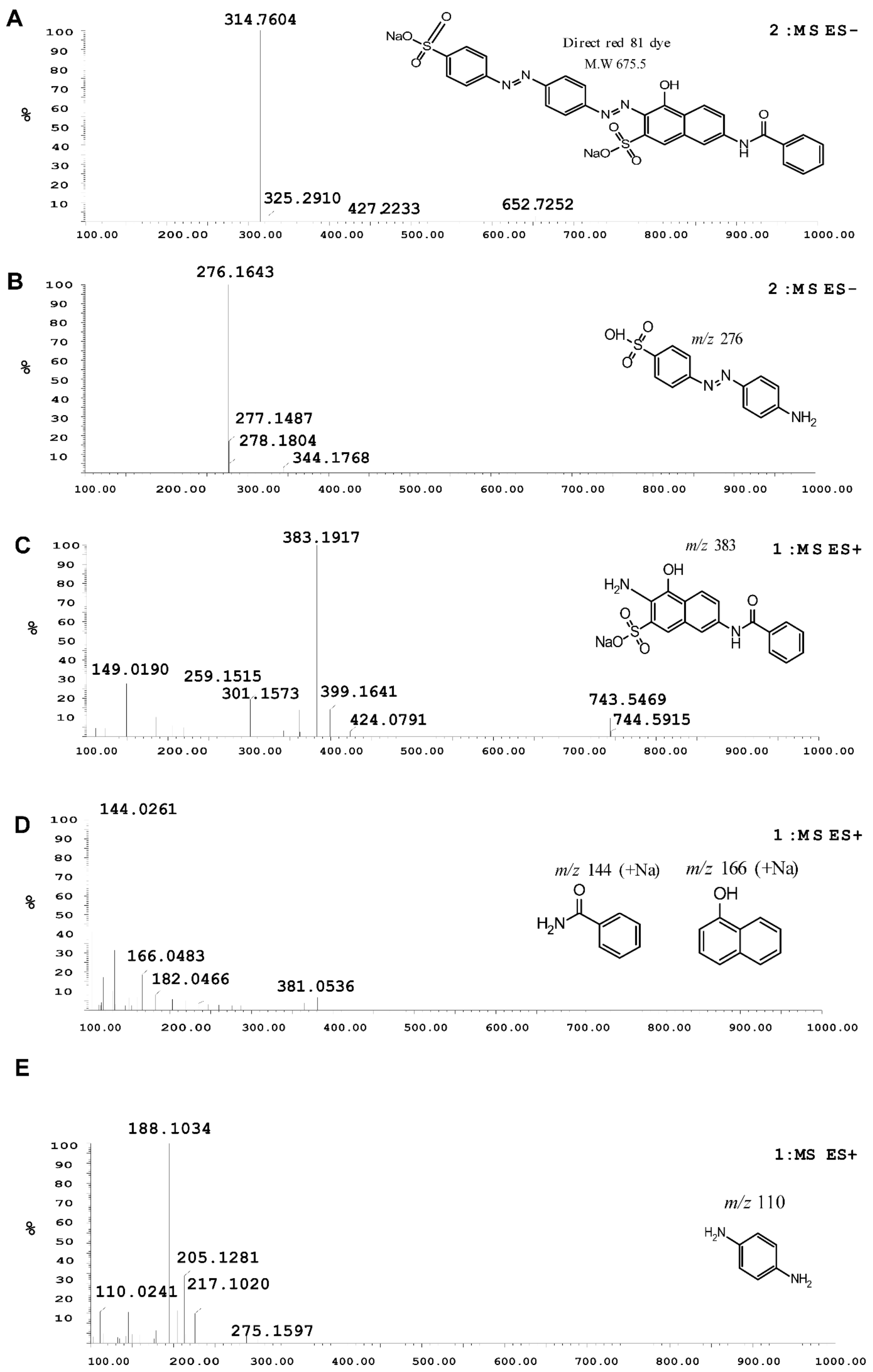

A
314.7604
325.2910
427.2233
652.7252
2 :MS ES-
Direct red 81 dye
M.W 675.5
NaO
OH
NaO
N
H
%
B
276.1643
277.1487
278.1804
344.1768
2 :MS ES-
m/z 276
OH
NH2
C
383.1917
149.0190
259.1515
301.1573
399.1641
424.0791
743.5469
744.5915
1 :MS ES+
m/z 383
OH
H2N
NaO
N
H
D
144.0261
166.0483
182.0466
381.0536
1 :MS ES+
m/z 144 (+Na)
m/z 166 (+Na)
H2N
OH
E
188.1034
205.1281
217.1020
110.0241
275.1597
1:MS ES+
m/z 110
H2N
NH2
100.00
200.00
300.00
400.00
500.00
600.00
700.00
800.00
900.00
1000.00

Figure S3. Mass spectra, chemical structures, and IUPAC names for the parent dye DR81 and the possible detected metabolites in the resolubilized methanolic extract of culture supernatant from symmetric cleavage by azoreductase extracted after 1 and 2 h incubation, analyzed using HPLC-MS. (**A**) DR81 parent dye with *m*/*z* 314.7 and 652.7 in the ESI- mode (sodium 7-benzamido-4-hydroxy-3-((E)-(4-((E)-(4-sulfonatophenyl)diazenyl)phenyl)diazenyl)naphthalene-2-sulfonate). (**B**) Metabolite with *m*/*z* 276 in the ESI- mode (E)-4-((4-aminophenyl)diazenyl)benzenesulfonic acid. (**C**) Metabolite with *m*/*z* 383 in the ESI+ mode (sodium 3-amino-7-benzamido-4-hydroxynaphthalene-2-sulfonate). (**D**) Metabolites in the ESI+ mode with *m*/*z* 166 (naphthalen-1-ol) and metabolite with *m*/*z* 144 (benzamide). (**E**) Metabolite with *m*/*z* 110 in the ESI+ mode (benzene-1,4-diamine).

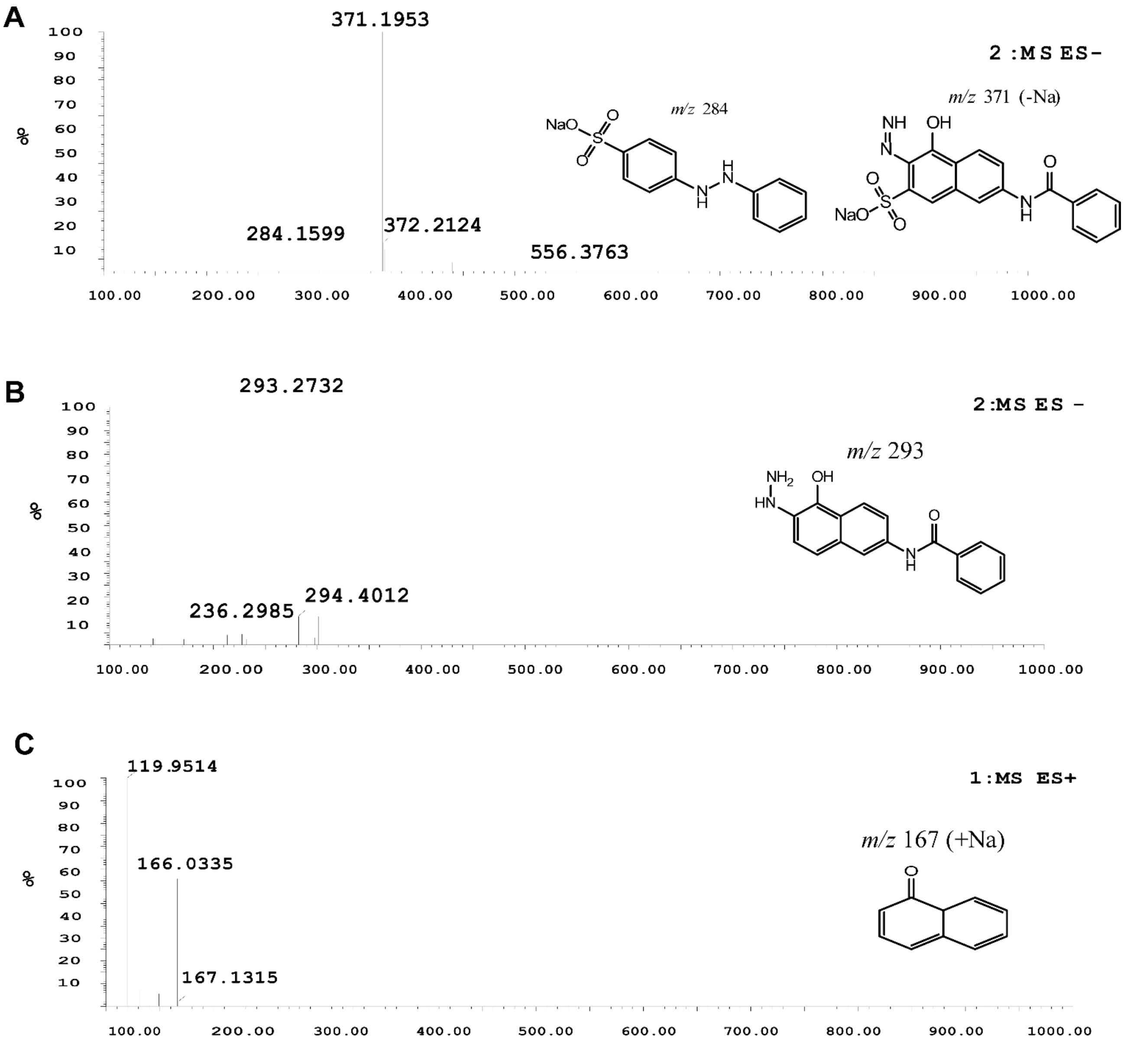

Figure S4. Mass spectra, chemical structures, and IUPAC names for the possible detected metabolites in the resolubilized methanolic extract of culture supernatant from assymmetric reductive cleavage extracted after 1 and 2 h incubation, analyzed using HPLC-MS. (**A**) Metabolites in the ESI- mode with *m*/*z* 371 (sodium 7-benzamido-3-diazenyl-4-hydroxynaphthalene-2-sulfonate) and *m*/*z* 284 (sodium 4-(2-phenylhydrazinyl)benzenesulfonate). (**B**) Metabolite with *m*/*z* 293 in the ESI- mode N-(6-hydrazinyl-5-hydroxynaphthalen-2-yl)benzamide. (**C**) Metabolite with *m*/*z* 167 in the ESI+ mode (naphthalen-1(8aH)-one).

Supplementary Tables

Table S1. Physicochemical parameters of non-sterile wastewater (NSWW) effluent from tanning industrial zone before treatment and after 8 h treatment with selected mixed cultures.

Parameters	Untreated wastewater at 0 time	Treated wastewater after 8 h by se- ected mixed cultures		
		27W	28W	34W
pH	8.94	8.76	8.68	8.6
TDS[1]	1450	1150	1050	1035
TSS[2]	88	56	50	43
COD[3]	2842	694	595	505
BOD[4]	500	50	45	41
Phosphate[5]	60	16.3	15.9	13.8
Ammonium	10	< 0.01	< 0.01	< 0.01
Nitrate	0.6	0.3	0.25	0.25
Total nitrogen	0.13	0.07	0.05	0.05
Phenols	3.2	1.88	1.5	1.38
Carbonate	nil	nil	nil	nil
Bicarbonate	976	10.67	15	10
Total hardness	740	680	590	501

[1] TDS: total dissolved solids,[2]TSS total suspended solids, [3]COD chemical oxygen demand, [4]BOD biological oxygen demand. [5]All parameters were expressed in mg/l, except pH.

Table S2: Primers used for PCR and sequencing of azoreductase genes in the selected mixed cultures

Name	Primer Sequence (5-3')	Microorganism	Product size (bp)	Annealing temp (˚C)
Alc_F_1	ATGAAACTGCTACACGTAGA	*Alcaligenes faecalis*	617	49.1
Alc_R_601	CTCTTTTCCAGCAAGGC			
Pch_F_29	ACACGGGCAAGGGTCTG	*Pseudochrobactrum saccharolyticum*	524	47.7
Pch_R_533	GTTCTCGTTTTTGCCGGATC			
Ach_F_1_8	ATGAAGACCCTCGTCATCC	*Achromobacter xylosoxidans*	626	51.2
Ach_R_612_8	GCCTGCCGCGAGACT			
Sten_F_1 [a]	ATGAAGCTTCTGCAYMTCGA	*Stenotrophomonas maltophilia*	574	50.2
Sten_R_558	CTTCGACCACTTCGTGC			
Ps_F_61 [a]	RCTCATGTTCAGGCC	*Pseudomonas monteilii*	505	49.1
Ps_R_549	CCAGCAGGATTCCGTTT			
Ps_F_18	CGATTCGAGCATCCTGG	*Pseudomonas putida*	583	49.1
Ps_R_583	AAACAGCTCGTTGGCAAT			
Ps_F_1 [a]	ATGAAACTGYTGYACATCGA	*Pseudomonas taiwanensis*	593	51.2
Ps_R_574 [a]	KCGYTGGCAATCTGCGCCTG			
Pr_F_1	ATGAAAAAGATCCTTGTACT	*Proteus vulgaris*	592	42
Pr_R_573 [a]	CAGYATCAATTTCTTTTTGT			
Paen_F_22	ACTGCCCATCCTCATGATCA	*Paenibacillus taichungensis*	518	44.6
Paen_R_520	ACAAAAATGCCTTCAAGCTT			
U4	AGG CCC GGG AAC GTA TTC AC	16S *rRNA* gene	481	52
U5T	TCA AAT GAA TTG ACG GGG GC			

[a] Degenerate primer

Table S3. Decolorization of DR81 azo dye by selected bacterial mixed cultures showing both decolorized suspensions and cell pellets.

**Azo dye**	**Mixed cul- ures**	**Before decolori- ation**	**After decolorization**	
			**Suspension**	**Supernatant nd cell pellets**
**Direct red 81**	27W	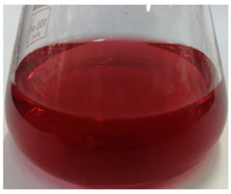	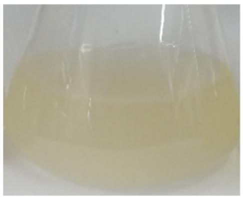	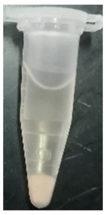
	28W	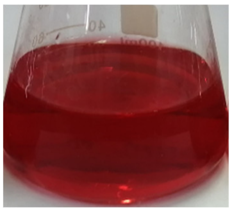	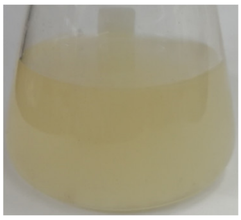	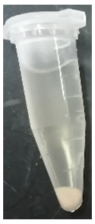
	34W	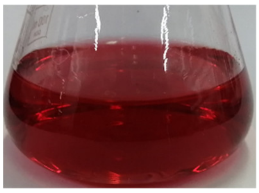	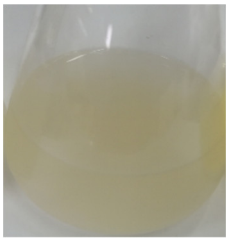	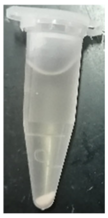
